# Effectiveness of mindfulness-based stress reduction in managing fatigue, anxiety, and depression among non-small cell lung cancer patients receiving surgery and adjuvant chemotherapy: A randomized controlled trial

**DOI:** 10.1097/MD.0000000000048134

**Published:** 2026-04-03

**Authors:** Xiaoqian Liu, Hui Chen, Yuna Cheng, Xinyi Xu, Haobo Shixing, Miao He, Yiqing Luo

**Affiliations:** a Department of Radiotherapy, Shanghai Chest Hospital, Shanghai Jiao Tong University School of Medicine, Shanghai, China; b Department of Thoracic Surgery, Shanghai Chest Hospital, Shanghai Jiao Tong University School of Medicine, Shanghai, China; c Department of Oncological Surgery, Shanghai Chest Hospital, Shanghai Jiao Tong University School of Medicine, Shanghai, China.

**Keywords:** anxiety, cancer-related fatigue, depression, mindfulness-based stress reduction, non-small cell lung cancer, randomized controlled trial, self-efficacy

## Abstract

**Background:**

Non-small cell lung cancer (NSCLC) patients undergoing surgery and adjuvant chemotherapy often experience debilitating physical and psychological symptoms, including cancer-related fatigue, anxiety, and depression. Mindfulness-based stress reduction (MBSR) has emerged as a complementary intervention to alleviate these burdens and enhance quality of life, but evidence in NSCLC populations remains limited.

**Methods:**

This randomized controlled trial enrolled 80 NSCLC patients, evenly divided into an MBSR group and a Control group. The MBSR group underwent an 8-week mindfulness program, while the Control group received standard care. Outcomes, including fatigue (Piper Fatigue Scale), anxiety (Self-Rating Anxiety Scale), depression (Self-Rating Depression Scale), self-efficacy (General Self-Efficacy Scale), and mindfulness awareness (Mindful Attention Awareness Scale), were assessed at 4 time points: baseline (before surgery), at the fourth week (before chemotherapy), and at 1 and 3 months post-intervention. Statistical analyses included repeated-measures ANOVA and *t* tests to evaluate group differences.

**Results:**

The MBSR group demonstrated significant improvements across all outcomes compared to the Control group. Fatigue levels in the MBSR group peaked at the fourth week but returned close to baseline levels by 3 months post-intervention, while the Control group maintained elevated fatigue scores (*P* < .05). Anxiety (Self-Rating Anxiety Scale) and depression (Self-Rating Depression Scale) scores significantly decreased in the MBSR group by the fourth week and continued to improve at subsequent time points (*P* < .01). Self-efficacy (General Self-Efficacy Scale) and mindfulness awareness (Mindful Attention Awareness Scale) showed consistent improvements in the MBSR group, with significant differences evident at 1 and 3 months post-intervention (*P* < .01).

**Conclusions:**

Mindfulness-based stress reduction effectively reduced fatigue, anxiety, and depression while enhancing self-efficacy and mindfulness awareness in NSCLC patients undergoing surgery and chemotherapy. These findings support the integration of mindfulness-based interventions into routine care to improve patient well-being during and after treatment.

## 1. Introduction

Lung cancer remains the leading cause of cancer-related deaths worldwide, accounting for a significant proportion of global cancer mortality. Among its subtypes, non-small cell lung cancer (NSCLC) represents approximately 85% of cases.^[1,2]^ Despite advancements in screening and treatment modalities, including surgical resection and adjuvant therapies, the prognosis for NSCLC patients remains poor, with high rates of morbidity and a 5-year survival rate under 25% for advanced stages.^[3]^ Adjuvant chemotherapy, often combined with immunotherapies, plays a pivotal role in improving outcomes; however, these treatments frequently exacerbate symptom burdens, including fatigue, anxiety, and depression, which significantly impact quality of life.^[4]^

Cancer-related fatigue (CRF) is among the most distressing and underrecognized symptoms in NSCLC patients undergoing treatment.^[5,6]^ Affecting up to 90% of patients, CRF is multifactorial, influenced by disease progression, treatment side effects, and psychosocial stressors.^[7]^ Persistent fatigue negatively affects recovery and overall well-being, with limited effective interventions available in standard clinical practice. Complementary approaches, such as physical activity and stress management, have shown promise, but their adoption in lung cancer care is still emerging.^[8]^ Thus, exploring alternative, patient-centered interventions like mindfulness-based therapies has gained traction in recent years.

Mindfulness-based interventions, including mindfulness-based stress reduction (MBSR), focus on cultivating present-moment awareness to manage stress and enhance emotional resilience.^[9]^ These therapies have shown efficacy in alleviating fatigue, anxiety, and depression in various cancer populations, including those with lung cancer.^[10]^ However, evidence specifically targeting NSCLC patients undergoing surgery and adjuvant chemotherapy remains sparse. This study aims to evaluate the effectiveness of MBSR in improving physical and psychological outcomes in this population, addressing critical gaps in supportive care for lung cancer patients.

## 2. Materials and methods

### 2.1. Study design

**Figure 1.**
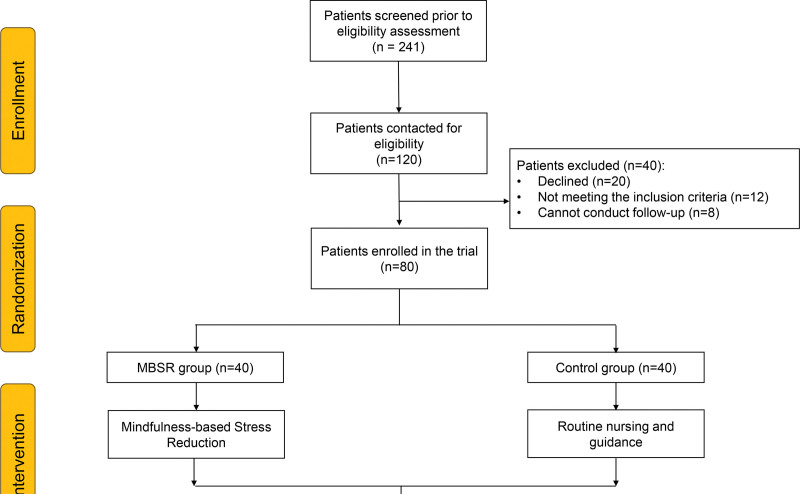
Flowchart. GSES = General Self-Efficacy Scale, MAAS = Mindful Attention Awareness Scale, MBSR = mindfulness-based stress reduction, PFS = Piper Fatigue Scale, SAS = Self-Rating Anxiety Scale, SDS = Self-Rating Depression Scale.

This study was a randomized controlled trial conducted to evaluate the effectiveness of MBSR interventions in improving cancer-related fatigue, anxiety, depression, self-efficacy, and mindfulness in postoperative NSCLC patients undergoing adjuvant chemotherapy. Participants were randomly assigned to either the MBSR group or the Control group in a 1:1 ratio, with 40 participants in each group (Fig. 1).

### 2.2. Participants

Inclusion criteria: Participants were eligible if they had a confirmed diagnosis of lung cancer through cytological, histological, or pathological examination; had undergone surgery and at least 1 session of chemotherapy within 1 month post-surgery; met the diagnostic criteria for cancer-related fatigue as per ICD-10 (International Classification of Diseases, 10th revision) guidelines; were aged 60 years or older; were literate and able to independently complete the questionnaire or complete it without cognitive impairment when provided with assistance; and provided informed consent and were willing to participate.

Exclusion criteria: Participants were excluded if they had tumor recurrence, metastasis, or multiple cancers; were in critical condition or unable to complete the study; had severe comorbidities affecting the heart, liver, kidneys, hematopoietic system, or infectious diseases; were in another clinical trial within the past 3 months or undergoing psychological or fatigue interventions.

### 2.3. Sample size

Sample size calculations were based on cancer-related fatigue as the primary outcome. Using data from a prior MBSR study, Piper Fatigue Scale (PFS) scores for the intervention and control groups were estimated at (5.0 ± 1.0) and (6.0 ± 1.0), respectively. The formula for comparing 2 means determined a minimum of 32 participants per group. To account for potential dropouts, the sample size was increased by 20%, resulting in 40 participants per group and a total of 80.

### 2.4. Randomization

Randomization was performed by an independent research assistant not involved in participant recruitment or data collection. The coin flip method was used to assign participants to the MBSR or Control group in a 1:1 ratio. To ensure allocation concealment, results of the randomization were placed in sealed opaque envelopes that were sequentially numbered and opened only after participant enrollment. While this method was practical, we acknowledge that it may introduce potential selection bias. Future studies could consider more sophisticated randomization techniques to minimize such bias.

### 2.5. Interventions

Control group: Participants received standard care, including routine hospital evaluations, health education on lung disease management, chemotherapy-related guidance, breathing exercises, and psychological support. Follow-ups included dietary advice, symptom management, and lifestyle recommendations.

MBSR group: Participants underwent an 8-week MBSR program led by certified trainers (Table 1). The intervention included mindfulness meditation, breathing techniques, and body scans. Training involved both in-person and home-based sessions supported by audio recordings and weekly phone calls for guidance. Participants logged their daily practice in a workbook.

**Table 1:** Intervention plan for cancer-related fatigue in elderly lung cancer patients post-surgery chemotherapy using mindfulness meditation.

Intervention strategy	1.Find a quiet and comfortable space: Ensure that the environment is peaceful and free from disturbances 2.Choose a comfortable posture: You may sit on a chair, lean against a cushion, or lie on a bed. Relax your muscles and make sure you’re comfortable 3.Close your eyes and begin breathing meditation: Focus your attention on your breathing. Pay attention to the sensations of air entering and leaving your body 4.Handling distractions: If your attention is interrupted by external factors, do not try to push them away. Instead, acknowledge them and allow them to gradually fade 5.Embrace thought and emotion fluctuations: During the meditation, if you experience fluctuations in thoughts or emotions, do not resist or attempt to change them. Let them flow naturally 6.Duration of meditation: The session typically lasts around 20 min. When finishing, slowly open your eyes and gently adjust your breath and posture 7.Optional use of soothing music: You may listen to calming music during the session to help relax your mind and deepen your meditation
Weekly schedule	Week 1: Beginner’s mind: • Approach the practice with a fresh perspective, allowing your mind and body to relax Week 2: Body scan: • Cultivate an “awakened” state rather than a “sleepy” one, focusing attention on your whole body to achieve a deeply relaxed state Week 3: Seated meditation: • Focus on your body while practicing seated meditation to reach a deep state of relaxation Week 4: Mindful yoga in a lying position: • Enhance flexibility, balance, and endurance through mindful yoga practice, focusing on each movement and breath
Intervention frequency	Participants will engage in this practice twice a week, using breathing meditation techniques
Duration of each session	Each session will last for 20 min
Timing of intervention	The intervention begins on the first day of the chemotherapy cycle and continues until the completion of chemotherapy
Location of intervention	During chemotherapy (1–2 h), with stable symptoms, participants will receive 1-on-1 instruction and guidance. Later, patients will be guided to practice self-directed mindfulness meditation at home
Mode of intervention	Participants will practice independently without interaction with others to maintain concentration and focus
Supplementary therapy	Gentle, relaxing music can be used during the session to promote relaxation and alleviate emotional tension

### 2.6. Primary outcome

The primary outcome was assessed using the PFS, developed by Piper in 1987.^[11]^ The scale evaluates CRF across 4 dimensions: behavioral, emotional, sensory, and cognitive. It comprises 22 items, with scores ranging from 0 to 10, where 0 indicates “no fatigue” and 10 represents “severe fatigue.” The total fatigue score is calculated as the mean of the 4 dimensions, categorized into mild (1–3), moderate (4–6), and severe (7–10) fatigue. The scale also includes an additional item measuring the duration of fatigue. The PFS has been validated for reliability, with a Cronbach α of 0.91 for the total scale and above 0.90 for all 4 dimensions. The Chinese version of the PFS has demonstrated excellent test-retest reliability (0.98), making it a robust tool for evaluating fatigue in clinical research.

### 2.7. Secondary outcomes

1. Self-Rating Anxiety Scale (SAS): This scale measures the severity of anxiety symptoms and treatment-related changes.^[12]^ It consists of 20 items, with responses scored on a 4-point Likert scale ranging from 1 (“none” or “occasional”) to 4 (“always”). Five positively worded items are reverse-scored. Total scores are categorized as follows: below 50 indicates no anxiety, 50 to 59 suggests mild anxiety, 60 to 69 moderate anxiety, and 70 or above indicates severe anxiety. The SAS has been widely validated, demonstrating strong reliability and sensitivity in clinical populations.
2. Self-Rating Depression Scale (SDS): This scale evaluates depressive symptoms across psychological, physical, and behavioral domains.^[13]^ It consists of 20 items, with positive items reverse-scored. Responses are rated on a 4-point Likert scale, and higher scores indicate greater severity. Scores are categorized as mild, moderate, or severe depression. The SDS has demonstrated satisfactory internal consistency (Cronbach α = 0.759) and is widely used to assess treatment efficacy in depressive conditions.
3. General Self-Efficacy Scale (GSES): The GSES is a unidimensional tool with 10 items assessing confidence in managing challenges and achieving goals.^[14]^ Each item is rated on a 4-point Likert scale, with higher scores indicating stronger self-efficacy. Scores are classified into 3 levels: high (≥80%), medium (60–80%), and low (≤60%). The GSES is validated for use in diverse populations, with a Cronbach α ranging from 0.75 to 0.94 and test-retest reliability of 0.55 to 0.75.
4. Mindful Attention Awareness Scale (MAAS): The MAAS measures mindfulness as a single dimension.^[15]^ It consists of 15 items rated on a 6-point Likert scale, from 1 (“always”) to 6 (“never”). Total scores range from 15 to 90, with higher scores reflecting greater mindfulness. Scores are classified into high (66–90), medium (41–65), and low (<40) mindfulness levels. The Chinese version of the MAAS has been validated and widely used for mindfulness assessment in clinical and research contexts.

### 2.8. Data collection and analysis

Data were collected at baseline (before surgery), at the fourth week (before chemotherapy), and at 1 and 3 months post-intervention (chemotherapy). Follow-up was conducted through in-person visits and telephone check-ins. Participants were asked to complete the outcome measures at each time point and were reminded about the assessments through weekly phone calls.

Statistical analyses included independent *t* tests, paired *t* tests, and repeated-measures ANOVA to evaluate within-group and between-group differences. Wilcoxon tests were used for nonparametric comparisons. All analyses were conducted using SPSS 24.0 (IBM Corp., Armonk), with a significance threshold of *P* < .05.

### 2.9. Ethical considerations

The study was approved by the Ethics Committee of Shanghai Chest Hospital (KS23020). Written informed consent was obtained from all participants, ensuring confidentiality and the right to withdraw at any time without repercussions.

## 3. Results

### 3.1. Participant flow and study design

The study screened 241 patients for eligibility, with 120 contacted for further assessment. Out of these, 80 patients met the inclusion criteria and were randomized into 2 groups: the MBSR group (n = 40) and the Control group (n = 40). The MBSR group underwent an 8-week mindfulness intervention, while the control group received routine nursing and guidance. Baseline characteristics and perioperative data, including measures of fatigue (PFS), anxiety (SAS), depression (SDS), self-efficacy (GSES), and mindfulness awareness (MAAS), were collected for evaluation. The inclusion and randomization process ensured a robust comparison between mindfulness-based intervention and routine care in managing perioperative outcomes. Notably, 40 patients were excluded due to declining participation (n = 20), failing inclusion criteria (n = 12), or inability to commit to follow-up (n = 8). This ensured the homogeneity and reliability of the final sample.

### 3.2. Baseline demographic and clinical characteristics

The baseline characteristics of the MBSR and Control groups were largely comparable, indicating successful randomization. Both groups had similar mean ages (Control: 69.67 years, MBSR: 70.72 years, *P* = .428) and gender distribution, with an equal proportion of males and females (*P* = 1.000). Physical metrics such as height, weight, and body mass index showed no significant differences, ensuring the groups were well-matched in terms of physical attributes.

While not statistically significant, the MBSR group had a higher proportion of current smokers (32.5%) compared to the Control group (12.5%, *P* = .079), which could influence outcomes. Comorbidities such as chronic obstructive pulmonary disease, diabetes, and hypertension were evenly distributed, with no notable differences. Similarly, the histological subtypes of lung cancer and tumor-node-metastasis staging were comparable, though the MBSR group included slightly more stage IV cases (10.0% vs 2.5%, *P* = .183).

Surgical interventions were balanced, with lobectomy being most common in both groups (Control: 60.0%, MBSR: 75.0%, *P* = .066). Preoperative pulmonary function tests also showed no significant differences, confirming baseline respiratory function was comparable. Overall, the groups were well-matched, with no significant differences that could bias the study outcomes (Table 2).

**Table 2:** Baseline characteristics and perioperative data of the MBSR group and Control group.

n	Level	Control (n = 40)	MBSR (n = 40)	*P*	SMD
Age, mean (SD)	69.67 (6.20)	70.72 (5.57)	0.428	.178	
Gender (%)	Female	20 (50.0)	19 (47.5)	1	0.05
	Male	20 (50.0)	21 (52.5)		
Height, mean (SD)		168.88 (10.01)	170.48 (9.75)	.471	0.162
Weight, mean (SD)		71.83 (11.75)	69.35 (12.24)	.359	0.206
BMI, mean (SD)		25.34 (3.51)	24.07 (3.99)	.134	0.339
Smoking (%)	Current-smoker	5 (12.5)	13 (32.5)	.079	0.521
	Ex-smoker	12 (30.0)	7 (17.5)		
	Never-smoker	23 (57.5)	20 (50.0)		
COPD (%)	No	31 (77.5)	30 (75.0)	1	0.059
	Yes	9 (22.5)	10 (25.0)		
Asthma (%)	No	39 (97.5)	38 (95.0)	1	0.132
	Yes	1 (2.5)	2 (5.0)		
IPF (%)	No	40 (100.0)	38 (95.0)	.474	0.324
	Yes	0 (0.0)	2 (5.0)		
Hypertension (%)	No	25 (62.5)	27 (67.5)	.815	0.105
	Yes	15 (37.5)	13 (32.5)		
Diabetes (%)	No	35 (87.5)	31 (77.5)	.377	0.265
	Yes	5 (12.5)	9 (22.5)		
Coronary artery disease (%)	No	35 (87.5)	37 (92.5)	.709	0.167
	Yes	5 (12.5)	3 (7.5)		
Cerebrovascular (%)	No	36 (90.0)	37 (92.5)	1	0.089
	Yes	4 (10.0)	3 (7.5)		
Histology (%)	Adenocarcinoma	14 (35.0)	21 (52.5)	.287	0.359
	Other	12 (30.0)	9 (22.5)		
	Squamous cell carcinoma	14 (35.0)	10 (25.0)		
TNM stage (%)	I	3 (7.5)	0 (0.0)	.183	0.508
	II	20 (50.0)	21 (52.5)		
	III	16 (40.0)	15 (37.5)		
	IV	1 (2.5)	4 (10.0)		
Adjuvant therapy (%)	Chemo	20 (50.0)	12 (30.0)	.127	0.467
	Chemo + immuno	14 (35.0)	16 (40.0)		
	Chemo + TKI	6 (15.0)	12 (30.0)		
Surgery type (%)	Lobectomy	24 (60.0)	30 (75.0)	.066	0.629
	Pneumonectomy	3 (7.5)	0 (0.0)		
	Segmentectomy	11 (27.5)	5 (12.5)		
	Wedgeresection	2 (5.0)	5 (12.5)		
Surgical approach (%)	Open	1 (2.5)	0 (0.0)	1	0.226
	VATS	39 (97.5)	40 (100.0)		
Preop FEV1/FVC, mean (SD)		64.03 (10.20)	62.70 (9.29)	.546	0.136
Preop FEV1 predicted percentage, mean (SD)		79.60 (15.82)	78.67 (16.45)	.799	0.057
Preop FVC predicted percentage, mean (SD)		87.46 (15.10)	83.74 (12.88)	.239	0.265
Preop DLCO percentage, mean (SD)		77.92 (12.89)	75.47 (14.22)	.423	0.18

BMI = body mass index, Chemo = chemotherapy, COPD = chronic obstructive pulmonary disease, DLCO = diffusing capacity of the lung for carbon monoxide, FEV1 = forced expiratory volume in 1 second, FVC = forced vital capacity, IPF = interstitial pulmonary fibrosis, Preop = preoperative, SD = standard deviation, SMD = standardized mean difference, TKI = tyrosine kinase inhibitor, TNM = tumor-node-metastasis, VATS = video-assisted thoracoscopic surgery.

### 3.3. Changes in fatigue over time between groups

**Figure 2.**
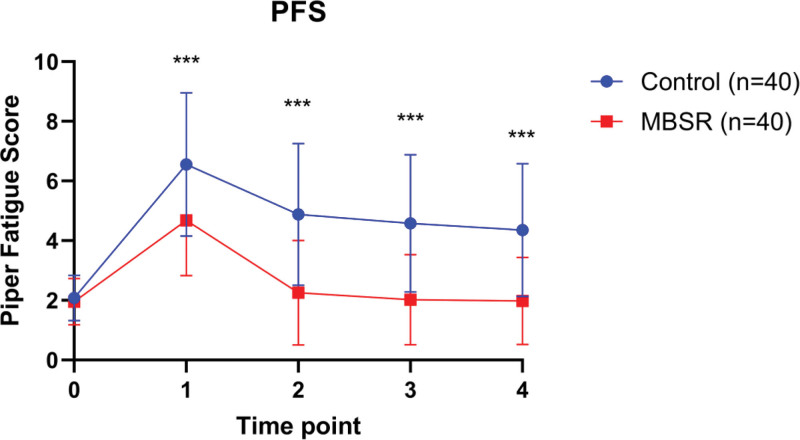
Changes in fatigue levels across 4 time points in the MBSR group and Control group. Data are presented as mean ± standard deviation. Blue circles represent the MBSR group, and red squares represent the Control group. ***P < .001. MBSR = mindfulness-based stress reduction, PFS = Piper Fatigue Scale.

Figure 2 illustrates the changes in fatigue levels, measured by the Patient Fatigue Scale (PFS), across 4 time points. The MBSR group demonstrated a significant reduction in fatigue compared to the Control group over time. Fatigue levels initially increased in both groups by the fourth week (before chemotherapy); however, this rise was notably less pronounced in the MBSR group. At 1 and 3 months post-intervention, the MBSR group exhibited a steady decline in fatigue scores, approaching baseline levels, whereas the Control group showed persistently higher fatigue scores.

By 3 months post-intervention, the divergence between the 2 groups was prominent, with the MBSR group maintaining significantly lower fatigue levels. These findings suggest that the MBSR intervention effectively mitigated fatigue associated with both surgery and chemotherapy, offering a sustainable improvement in physical well-being.

### 3.4. Psychological and behavioral outcomes over time

**Figure 3.**
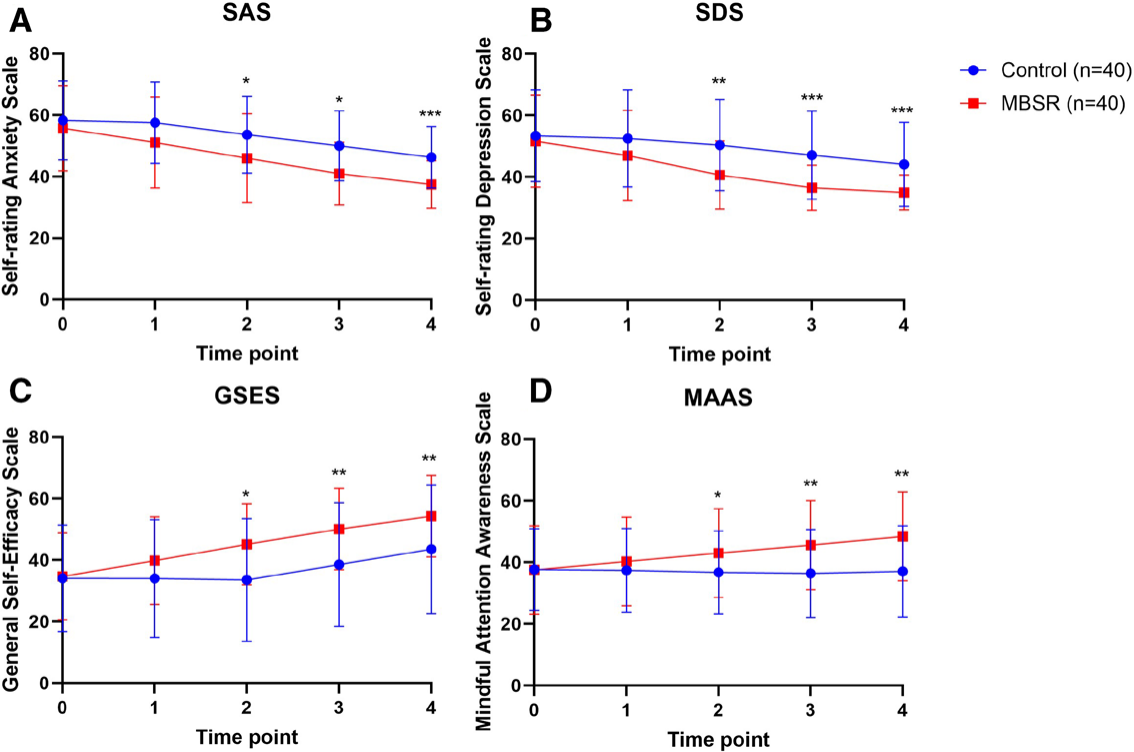
Changes in psychological and behavioral outcomes over 4 time points for the MBSR and Control groups. (A) Self-Rating Anxiety Scale (SAS): Anxiety levels decreased significantly in the MBSR group compared to the Control group (*P < .05, ***P < .001). (B) Self-Rating Depression Scale (SDS): Depression scores showed significant reductions in the MBSR group over time (**P < .01, ***P < .001). (C) General Self-Efficacy Scale (GSES): The MBSR group exhibited improved self-efficacy compared to the Control group (*P < .05, **P < .01). (D) Mindful Attention Awareness Scale (MAAS): Mindfulness awareness scores increased significantly in the MBSR group (*P < .05, **P < .01). Data are shown as mean ± standard deviation, with blue circles representing the Control group and red squares representing the MBSR group. GSES = General Self-Efficacy Scale, MAAS = Mindful Attention Awareness Scale, MBSR = mindfulness-based stress reduction, SAS = Self-Rating Anxiety Scale, SDS = Self-Rating Depression Scale.

Figure 3 illustrates changes in anxiety (SAS), depression (SDS), self-efficacy (GSES), and mindfulness awareness (MAAS) across 4 time points. The MBSR group exhibited significant improvements in all measured outcomes compared to the Control group. Anxiety (SAS) and depression (SDS) levels declined steadily in the MBSR group, with statistically significant differences (*P* < .01) emerging by the fourth week and becoming more pronounced at 1 and 3 months post-intervention.

Similarly, self-efficacy (GSES) and mindfulness awareness (MAAS) demonstrated consistent increases in the MBSR group across all time points. The differences between the groups were significant (*P* < .05 to *P* < .01) by 1 month post-intervention, with the MBSR group showing marked enhancements in their psychological resilience and mindfulness awareness. These results underscore the efficacy of MBSR in improving psychological and behavioral outcomes, even amidst the challenges posed by chemotherapy.

## 4. Discussion

This study demonstrates that MBSR significantly alleviates fatigue, anxiety, and depression while enhancing self-efficacy and mindfulness awareness in patients with NSCLC undergoing surgery and adjuvant chemotherapy. The intervention group showed substantial improvements in these outcomes compared to the control group, particularly in reducing CRF, one of the most debilitating symptoms experienced by lung cancer patients. These findings align with the growing body of evidence supporting the efficacy of MBSR as an adjunctive therapy for cancer care, offering a holistic approach to managing both psychological and physical burdens associated with treatment. Notably, our results showed that MBSR not only addressed acute symptom relief but also supported overall recovery, suggesting its potential integration into routine clinical practice.

Cancer-related fatigue, prevalent among lung cancer patients, often remains underdiagnosed and inadequately managed despite its profound impact on quality of life and treatment adherence.^[16,17]^ Our findings confirm earlier research that identified mindfulness-based interventions as effective in reducing CRF by promoting better emotional regulation and stress management. The structured 8-week MBSR program in our study aligns with similar interventions, demonstrating significant reductions in fatigue levels, corroborating the work of McDonnell et al^[18]^ and Li et al.^[19]^ These improvements likely stem from MBSR’s focus on cultivating present-moment awareness, which reduces the physiological and psychological strain associated with cancer treatment.^[20]^

The observed reductions in anxiety and depression are consistent with previous research that underscores the psychological benefits of MBSR. For instance, McDonnell et al^[10]^ and Li et al^[19]^ found that mindfulness interventions reduced psychological distress and improved emotional resilience in lung cancer patients. These improvements are particularly relevant for patients undergoing surgery and chemotherapy, who often face heightened stress levels. The integration of mindfulness practices, such as mindful breathing and meditation, has been shown to modulate the hypothalamic–pituitary–adrenal axis, reducing stress hormone levels and fostering a sense of calm.^[8,21]^ Additionally, mindfulness enhances self-awareness, allowing patients to better manage their emotional responses to the challenges posed by their diagnosis and treatment.^[22]^

Self-efficacy, a critical determinant of health behaviors and coping strategies, significantly improved in the MBSR group. Previous studies have identified self-efficacy as a mediator for adherence to treatment regimens and proactive health behaviors in cancer patients.^[23]^ The tailored nature of our MBSR program likely contributed to these improvements, as participants were equipped with practical tools to manage their symptoms independently. Moreover, increased mindfulness awareness, as reflected in improved Mindful Attention Awareness Scale scores, highlights the effectiveness of MBSR in fostering sustained psychological resilience. These findings align with the meta-analysis by Dong et al,^[24]^ which emphasized the role of structured mindfulness practices in enhancing psychological well-being in cancer patients. A comprehensive review of the literature shows that mindfulness-based interventions, such as MBSR, have demonstrated effectiveness in reducing CRF, anxiety, and depression across diverse populations, including both Western and Eastern cultures.^[10,25]^ Furthermore, studies on psychoeducational programs, such as the one conducted by Paça et al, also demonstrate significant benefits in reducing perceived stress and improving psychological well-being in cancer patients, which is aligned with our findings.^[26]^ Interestingly, the differential effects of mindfulness-based interventions in these cultures may be influenced by differences in societal stressors and coping mechanisms.

The adoption and adaptation of mindfulness practices may differ across cultural contexts. In Western cultures, mindfulness has been embraced primarily as a stress-management strategy, often aligned with individualistic values emphasizing self-regulation. In contrast, within Eastern societies, including China, mindfulness resonates with collectivist values and the pursuit of harmony between mind and body. These cultural distinctions influence both adherence and perceived benefits. Consistent with findings by Paça et al, social stressors and coping mechanisms embedded in cultural value systems shape the effectiveness of mindfulness-based interventions.^[26]^ The beneficial outcomes of MBSR may be explained by several interrelated biopsychosocial mechanisms. Neurophysiologically, mindfulness practices are known to modulate the hypothalamic–pituitary–adrenal (HPA) axis, reducing cortisol secretion and attenuating the stress response.^[27]^ Moreover, mindfulness can downregulate neuroinflammatory pathways by decreasing circulating pro-inflammatory cytokines and enhancing parasympathetic tone via vagal activation.^[28]^ These physiological adjustments contribute to improved emotional regulation and reduced fatigue. Psychologically, MBSR fosters adaptive coping and cognitive flexibility, enhancing self-efficacy and emotional resilience. Together, these pathways provide a plausible biopsychosocial explanation for the observed improvements in fatigue, anxiety, and depression.

Despite the promising results, our study has several limitations. First, the relatively small sample size and single-center design may limit the generalizability of the findings. While the randomized controlled design strengthens the validity of our conclusions, larger multicenter trials are necessary to confirm these results across diverse populations. Second, the reliance on self-reported measures introduces potential biases, emphasizing the need for objective biomarkers to validate outcomes. Third, while our study focused on short-term benefits, the long-term sustainability of MBSR’s effects remains unclear and warrants further investigation. Cultural and socioeconomic factors that may influence mindfulness practice adherence were not explored but represent an important avenue for future research, especially in regions with rising lung cancer prevalence.^[29]^ Last limitation of this study is the absence of blinding of participants, practitioners, and assessors, which could introduce potential performance and detection bias. Future studies should consider implementing assessor blinding or using objective physiological indicators to reduce this risk.

## 5. Conclusion

This study underscores the critical role of MBSR as a complementary therapy in the management of physical and psychological symptoms among NSCLC patients undergoing surgery and adjuvant chemotherapy. The integration of MBSR into oncology care has the potential to enhance patient-centered outcomes, bridging gaps in supportive care. By focusing on holistic strategies that target both mind and body, MBSR can contribute to improving the overall quality of life for cancer patients, aligning with the evolving emphasis on comprehensive, multidisciplinary treatment approaches in oncology.

Future research should prioritize large-scale, multicenter trials to validate these findings across diverse populations and settings. Exploring the long-term sustainability of MBSR’s benefits and identifying optimal delivery formats, such as digital or hybrid interventions, could expand accessibility for broader patient populations. Additionally, investigating the integration of MBSR with other supportive therapies, including exercise and nutritional interventions, may yield synergistic effects. Objective biomarkers, such as stress hormones and inflammatory markers, should be incorporated into future studies to provide physiological insights into the mechanisms underpinning MBSR’s effects. By addressing these avenues, future research can advance the development of tailored, evidence-based interventions that support holistic recovery and resilience in cancer care.

## Author contributions

**Conceptualization:** Xiaoqian Liu, Yiqing Luo.

**Data curation:** Hui Chen, Haobo Shixing.

**Formal analysis:** Xiaoqian Liu, Haobo Shixing.

**Funding acquisition:** Hui Chen.

**Investigation:** Xiaoqian Liu, Hui Chen, Xinyi Xu, Miao He.

**Methodology:** Xiaoqian Liu, Yuna Cheng.

**Project administration:** Yiqing Luo.

**Resources:** Hui Chen, Xinyi Xu.

**Software:** Hui Chen, Yuna Cheng.

**Supervision:** Miao He, Yiqing Luo.

**Validation:** Hui Chen.

**Visualization:** Yiqing Luo.

**Writing** – **review & editing:** Miao He, Yiqing Luo.
